# Alcohol-Related Hospitalizations From 2016 to 2022

**DOI:** 10.1001/jamanetworkopen.2025.50589

**Published:** 2025-12-23

**Authors:** Eden Y. Bernstein, Linnea M. Wilson, Gina R. Kruse, E. Jennifer Edelman, Shoshana J. Herzig, Timothy S. Anderson

**Affiliations:** 1 Division of Hospital Medicine, Department of Medicine, University of Colorado School of Medicine, Aurora; 2 Division of General Internal Medicine, Beth Israel Deaconess Medical Center, Boston, Massachusetts; 3 Division of General Internal Medicine, Department of Medicine, University of Colorado School of Medicine, Aurora; 4 Department of Internal Medicine, Yale School of Medicine, New Haven, Connecticut; 5 Program in Addiction Medicine, Yale School of Medicine, New Haven, Connecticut; 6 Harvard Medical School, Boston, Massachusetts; 7 Division of General Internal Medicine, University of Pittsburgh, Pittsburgh, Pennsylvania; 8 Center for Health Equity Research and Promotion, VA Pittsburgh Health System, Pittsburgh, Pennsylvania

## Abstract

**Importance:**

Unhealthy alcohol use contributes to a high rate of mortality. While alcohol use increased during the COVID-19 pandemic, recent trends in alcohol-related hospitalizations are unknown.

**Objective:**

To examine US trends in the rate and outcomes of alcohol-related hospitalizations from 2016 to 2022.

**Design, Setting, and Participants:**

This serial cross-sectional study examined hospitalizations among adults aged 18 years or older who were sampled in the National Inpatient Sample, weighted to reflect nationally representative estimates. Data were examined from April to October 2025.

**Main Outcomes and Measures:**

The primary outcome was alcohol-related hospitalizations, defined using discharge diagnosis codes. Hospitalizations were categorized as primary alcohol use disorder (AUD), primary alcohol-related medical complication, and secondary alcohol-related diagnosis based on discharge diagnosis codes. Secondary outcomes included in-hospital mortality, length of stay, cost of hospitalizations, and discharge disposition.

**Results:**

This study included a weighted 12 912 240 alcohol-related hospitalizations (age 50 to 64 years, 40.4% [95% CI, 40.3%-40.5%]; male, 71.5% [95% CI, 71.4%-71.6%]; Black, 15.6% [95% CI, 15.3%-15.9%]; Hispanic, 11.2% [95% CI, 10.9%-11.4%]; White, 64.9% [95% CI, 64.5%-65.3%]). From 2016 to 2022, the annual rate of alcohol-related hospitalizations per 100 000 remained stable from 721 in 2016 to 688 in 2022 (annual percentage change [APC], −0.43; 95% CI, −1.28 to 0.49) but increased from 70 to 83 among hospitalizations for alcohol-related medical complications (APC, 3.56; 95% CI, 2.19 to 4.94). Trends across demographic groups differed by reasons for hospitalization. In-hospital mortality increased from 2.4% (95% CI, 2.3% to 2.5%) in 2016 to 3.1% (95% CI, 3.0% to 3.2%) in 2022 (*P* < .001). Mean length of stay increased from 5.6 (95% CI, 5.6 to 5.7) to 6.2 (95% CI, 6.1 to 6.3) days (*P* < .001), and the rate of self-directed discharges increased from 5.0% (95% CI, 4.8% to 5.2%) to 6.3% (95% CI, 6.1% to 6.5%) (*P* < .001). Hospitalization costs increased even after accounting for inflation and amounted to $32.6 billion in 2022.

**Conclusion and Relevance:**

In this serial cross-sectional study of nationally representative administrative data from 2016 and 2022, the rate of alcohol-related hospitalizations was stable while mortality, length of stay, and health care costs all increased. Preventive efforts are needed to improve outcomes and reduce health care spending by reducing population-level alcohol consumption and engaging patients in AUD treatment before progression to alcohol-related hospitalizations.

## Key Points

**Question:**

What are national trends in alcohol-related hospitalizations and outcomes?

**Findings:**

In this cross-sectional study of more than 12.9 million hospitalizations in the 2016 to 2022 National Inpatient Sample, the annual rate of alcohol-related hospitalizations per 100 000 was stable from 721 to 688 but increased for hospitalizations with primary diagnoses of alcohol-related medical complications from 70 to 83. Inpatient mortality rates, length of stay, patient complexity, and costs also increased during this period, contributing to $32.6 billion in health care costs in 2022.

**Meaning:**

These findings suggest that expanded alcohol-related treatment and prevention efforts are needed to improve patient outcomes and reduce health care spending from alcohol-related hospitalizations.

## Teaser

This cross-sectional study examines US trends in the rate and outcomes of alcohol-related hospitalization from 2016 to 2022 using data from the National Inpatient Sample.

## Introduction

More than 1 in 10 US adults have alcohol use disorder (AUD).^1^ Rates of alcohol-attributable mortality have doubled in recent decades^2^ and further increased during the COVID-19 pandemic, amounting to nearly 180 000 annual US deaths in 2021.^3^ AUD also contributed to nearly $30 billion in health care expenditures in 2010, driven mostly by specialty addiction care and hospitalizations.^4^ Compared with other substance use disorders (excluding tobacco), persons with AUD contribute the greatest share of hospitalizations,^5^ and alcohol-related hospitalizations incur the highest medical costs.^6^

Hospitalizations are also important because they signify high acuity of medical conditions and an opportunity to initiate evidence-based treatment for the 2 million US adults with AUD who are hospitalized each year.^5^ The 5-year mortality rate for hospitalized patients with AUD at the Veterans Health Administration is over 30%.^7^ While alcohol-related hospitalizations present an opportunity to engage a highly vulnerable population with treatment, less than 1% of hospitalized Medicare beneficiaries with AUD are prescribed evidence-based medications for AUD.^8^

A prior study using national data found that AUD hospitalizations rose by 3.5% from 1998 to 2016, while in-hospital mortality decreased by 25% during the same period.^9^ However, alcohol use has increased during the COVID-19 pandemic.^10^ During this period, rates of alcohol-related mortality,^11^ hospitalizations due to high-acuity alcohol-related conditions in a commercially insured sample,^12^ and alcohol-induced liver disease in a nationally representative sample have also increased.^13^ Recent rates of alcohol-related hospitalizations are unknown. Therefore, we sought to characterize more recent trends in alcohol-related hospitalizations using the largest available nationally representative sample of inpatient hospitalizations.

## Methods

We conducted a serial cross-sectional analysis using the 2016 to 2022 Healthcare Cost and Utilization Project (HCUP) National Inpatient Sample (NIS).^14^ NIS includes a stratified sample of 20% of all community hospital discharges in the US (excluding rehabilitation and long-term acute care hospitals) designed to produce nationally representative estimates of inpatient health care use. Because the NIS data used for these analyses are fully deidentified and were not collected solely for this study, this study was determined to be exempt from review by the Beth Isreal Deaconess Medical Center institutional review board and informed consent was not required. We followed the Strengthening the Reporting of Observational Studies in Epidemiology (STROBE) reporting guideline for cross-sectional studies.

### Population

We identified alcohol-related hospitalizations among adults age 18 years or older using *International Classification of Diseases, 10th Revision* (*ICD-10*) discharge diagnosis codes for AUD (alcohol use, abuse, or dependence, excluding in remission specifiers^15^) or alcohol-related medical complications defined using chronic 100% alcohol-attributable *ICD-10 *codes in the Center for Disease Control Alcohol-Related Disease Impact (eTable 1 in Supplement 1).^16^ Codes were included in both the primary and nonprimary positions because ordering is influenced by reimbursement, and it is possible for alcohol to be a proximal cause of hospitalizations even if coded in a nonprimary position.^17^ We further categorized hospitalizations into 3 mutually exclusive subgroups based on position of *ICD-10* codes: primary AUD, primary alcohol-related medical complication, and secondary alcohol-related diagnosis defined as hospitalizations that did not have either an AUD or alcohol-related medical complication diagnosis in the primary position.

### Outcomes

The primary outcome was rate of AUD hospitalizations. Secondary hospitalization outcomes included in-hospital mortality, length of stay, and costs. Costs reflect expenses incurred from hospital services. In NIS, they are estimated by converting charges (the amount a hospital bills for services) using hospital-level cost-to-charge ratios.^14^ Costs were reported per hospitalization and overall calculated as the sum of all hospitalization costs by calendar year. We accounted for inflation by adjusting costs to 2016 US dollars using the annual average Consumer Price Index from the Bureau of Labor Statistics.^18^ We used the Elixhauser index for readmissions as a proxy for patient complexity.^19^ Finally, discharge disposition included home, facility, self-directed discharge, or other.

### Additional Measures

We included the following demographic and socioeconomic variables: age, sex, race or ethnicity (derived from discharge records and categorized by NIS as Asian, non-Hispanic Black, Hispanic, Native American, non-Hispanic White, and other), primary payer, and quartile of median household income (by patient zip-code derived from annual census data). Other race included multiracial and any race or ethnicity not otherwise specified. We accounted for regional variation by including US Census divisions of hospitals and metropolitan status determined by urban-rural classifications of patient residence. Missing values were coded as a separate category.

### Statistical Analysis

We applied sampling weights, accounting for clusters and strata, to produce nationally representative estimates with accompanying measures of variance for all analyses in line with NIS methods specifications.^14^ We accounted for population shifts overall and across subgroups by calculating population-adjusted rates of hospitalizations using data from the US Census Bureau.^20^ First, we estimated trends in the total rate of alcohol-related hospitalizations from 2016 to 2022 in the overall cohort and by the 3 mutually exclusive diagnosis subgroups (primary AUD, primary alcohol-related medical complication, secondary alcohol-related diagnosis). We determined trends using joinpoint regressions which use permutation tests to identify significant changes in trends over time and provide annual percentage change (APC) and associated 95% CIs.^21^ Second, we repeated this analysis across subgroups of demographics (age, sex, race and ethnicity, census division, metropolitan residence) and economic factors (payer and median income). We excluded the other race and ethnicity category and no charge and other payer categories because they are not included in Census data. Third, we estimated trends in hospitalization outcomes of mortality, length of stay, cost, Elixhauser readmissions index, and discharge disposition. We used univariable regressions fitted to the specific outcomes (logistic regression for mortality and discharge disposition, negative binomial for length of stay, and linear for cost and Elixhauser) to determine statistical significance of trends from 2016 and 2022. Fourth, we repeated the trends analysis stratified by demographic and socioeconomic factors and the trends in outcomes across the 3 subgroups because they may vary by reason for hospitalization. We conducted all analyses using SAS 9.4 (SAS Institute) and the Joinpoint Regression Software version 5.4.0 (Joinpoint)^21^ and generated 95% CIs for population estimates to account for variance from the complex sampling design. Statistical significance was set at *P* < .05, and tests were 2-sided. Data were analyzed from April to Ocotber 2025.

## Results

We identified 2.6 million alcohol-related hospitalizations, representative of a weighted 12 912 240 (95% CI, 12 765 314 to 13 059 165) US hospitalizations after applying sampling weights. Overall, 40.4% (95% CI, 40.3% to 40.5%) were aged 50 to 64 years, 71.5% (95% CI, 71.4% to 71.6%) were male, 1.0% (95% CI, 1.0% to 1.1%) were Asian, 15.6% (95% CI, 15.3% to 15.9%) were Black, 11.2% (95% CI, 10.9% to 11.4%) were Hispanic, 1.6% (95% CI, 1.5% to 1.7%) were Native American, 64.9% (95% CI, 64.5% to 65.3%) were White, and 33.7% (95% CI, 33.4% to 34.0%) were insured by Medicaid (Table 1). Of the weighted 12 912 240 alcohol-related hospitalizations, 2 238 459 (95% CI, 2 189 419 to 2 287 499) had primary AUD, 1 432 014 (95% CI, 1 415 675 to 1 448 353) had primary alcohol-related medical complications, and 9 241 766 (95% CI, 9 138 598 to 9 344 935) had secondary alcohol-related diagnoses. Most primary alcohol-related medical complication diagnoses were liver disease and pancreatitis (eTable 2 in Supplement 1). Among secondary alcohol-related diagnosis hospitalizations, the most common primary diagnosis category in 2022 was psychiatric followed by circulatory, injury, and digestive (eTable 3 in Supplement 1). Primary psychiatric diagnoses represented the largest absolute change, decreasing from 283 545 in 2016 to 199 590 in 2022.

**Table 1.** Characteristics of US Patients With Alcohol-Related Hospitalizations, National Inpatient Sample 2016 to 2022

Characteristic	Cohort, % (95% CI)			
	Overall	Primary AUD	Primary alcohol-related medical complications	Secondary alcohol-related diagnosis
Unweighted, No.	2 582 449	447 692	286 403	1 848 354
Weighted, No. (95% CI)	12 912 240 (12 765 314-13 059 165)	2 238 459 (2 189 419-2 287 499)	1 432 014 (1 415 675-1 448 353)	9 241 766 (9 138 598-9 344 935)
Demographics				
Age, y				
18-34	13.9 (13.8-14.0)	17.6 (17.4-17.7)	14.0 (13.8-14.1)	13.1 (12.9-13.2)
35-49	25.9 (25.8-26.0)	35.2 (35.1-35.4)	33.9 (33.7-34.1)	22.4 (22.3-22.5)
50-64	40.4 (40.3-40.5)	37.7 (37.4-37.9)	40.7 (40.5-40.9)	41.1 (40.9-41.2)
≥65	19.7 (19.6-19.8)	9.5 (9.4-9.6)	11.5 (11.4-11.6)	23.4 (23.3-23.6)
Sex				
Female	28.5 (28.4-28.6)	26.3 (26.0-26.5)	32.1 (31.9-32.3)	28.4 (28.3-28.5)
Male	71.5 (71.4-71.6)	73.7 (73.5-73.9)	67.9 (67.7-68.1)	71.5 (71.4-71.6)
Race or ethnicity^a^				
Asian	1.0 (1.0-1.1)	0.9 (0.8-0.9)	1.3 (1.2-1.3)	1.0 (1.0-1.1)
Black	15.6 (15.3-15.9)	11.5 (11.0-12.0)	14.5 (14.2-14.7)	16.8 (16.5-17.1)
Hispanic	11.2 (10.9-11.4)	10.6 (10.3-11.0)	14.7 (14.3-15.0)	10.7 (10.5-11.0)
Native American	1.6 (1.5-1.7)	1.5 (1.4-1.6)	1.9 (1.7-2.0)	1.6 (1.5-1.8)
White	64.9 (64.5-65.3)	69.7 (68.9-70.5)	62.1 (61.7-62.6)	64.2 (63.8-64.6)
Other	2.7 (2.6-2.8)	2.9 (2.8-3.1)	2.9 (2.8-3.0)	2.6 (2.5-2.7)
Residence^b^				
Metropolitan	84.6 (84.3-84.8)	86.7 (86.4-87.1)	85.6 (85.3-85.8)	83.9 (83.6-84.1)
Nonmetropolitan	13.4 (13.1-13.6)	10.9 (10.5-11.2)	13.4 (13.2-13.7)	14.0 (13.7-14.2)
Socioeconomic groups				
Payer^c^				
Private	22.8 (22.5-23.0)	24.2 (23.7-24.7)	26.7 (26.4-27.0)	21.8 (21.6-22.0)
Medicare	28.3 (28.1-28.4)	17.6 (17.5-17.8)	19.5 (19.3-19.7)	32.2 (32.1-32.4)
Medicaid	33.7 (33.4-34.0)	40.9 (40.2-41.6)	37.0 (36.7-37.3)	31.5 (31.2-31.7)
Self	10.1 (9.9-10.3)	11.9 (11.6-12.2)	12.1 (11.9-12.3)	9.3 (9.2-9.5)
No charge	1.1 (1.0-1.1)	1.4 (1.2-1.5)	1.1 (1.0-1.1)	1.0 (0.9-1.0)
Other	3.9 (3.8-4.0)	3.7 (3.5-3.9)	3.5 (3.4-3.6)	4.0 (3.9-4.1)
Income percentile^d^				
0-25	32.6 (32.2-33.0)	27.9 (27.3-28.5)	31.4 (31.0-31.9)	33.9 (33.5-34.3)
26-50	25.1 (24.8-25.3)	24.1 (23.7-24.5)	25.6 (25.2-25.9)	25.2 (25.0-25.5)
51-75	22.1 (21.8-22.4)	23.4 (23.1-23.8)	23.4 (23.1-23.7)	21.6 (21.3-21.8)
76-100	16.8 (16.5-17.2)	21.0 (20.4-21.6)	17.4 (17.0-17.7)	15.8 (15.4-16.1)

Abbreviations: AUD, alcohol use disorder; NIS, national inpatient sample.

^a^ Race and ethnicity were missing for 2.97% (95% CI, 2.75%-3.20%) of the overall cohort. Other race includes multiracial and any race or ethnicity not otherwise specified.

^b^ Residence was missing for 2.07% (95% CI, 1.97%-2.17%) of the overall cohort.

^c^ Payer was missing for 3.87% (95% CI, 3.78%-3.97%) of the overall cohort.

^d^ Estimated income based on residential zip code was missing for 3.40% (95% CI, 3.30%-3.50%) of the overall cohort.

### Trends in Hospitalizations

**Figure 1.**
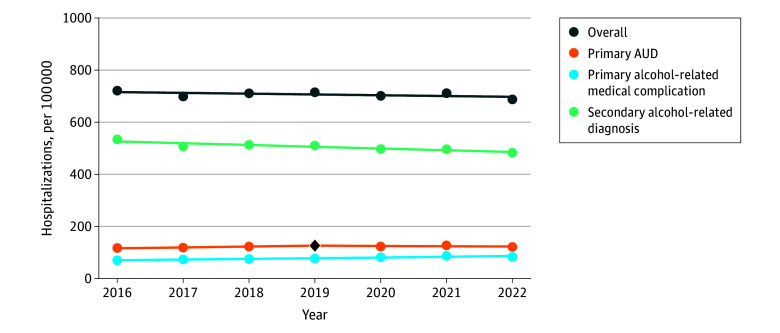
Trends in Rates of Alcohol-Related Hospitalizations From 2016 to 2022 Segments indicate annual percentage changes derived from joinpoint regressions. A single segement (0 joinpoints) indicates a consistent rate from 2016 to 2022, and a joinpoint indicated by a black diamond represents a change in direction or magnitude. AUD indicates alcohol use disorder.

Overall, annual rates of alcohol-related hospitalizations per 100 000 remained stable from 721 in 2016 to 688 in 2022 (APC, −0.43; 95% CI, −1.28 to 0.49) but increased from 70 to 83 for primary alcohol-related medical complication hospitalizations (APC, 3.56; 95% CI, 2.19 to 4.94) (Figure 1). Rates of primary AUD hospitalizations per 100 000 increased from 117 in 2016 to 127 in 2019 (APC, 2.77; 95% CI, 0.98 to 6.20) but remained stable from 2019 to 2022 (APC, −0.90; 95% CI, −3.68 to 0.84). Rates of secondary alcohol-related diagnosis hospitalizations per 100 000 decreased from 534 in 2016 to 483 in 2022 (APC, −1.33; 95% CI, −1.98 to −0.64).

**Figure 2.**
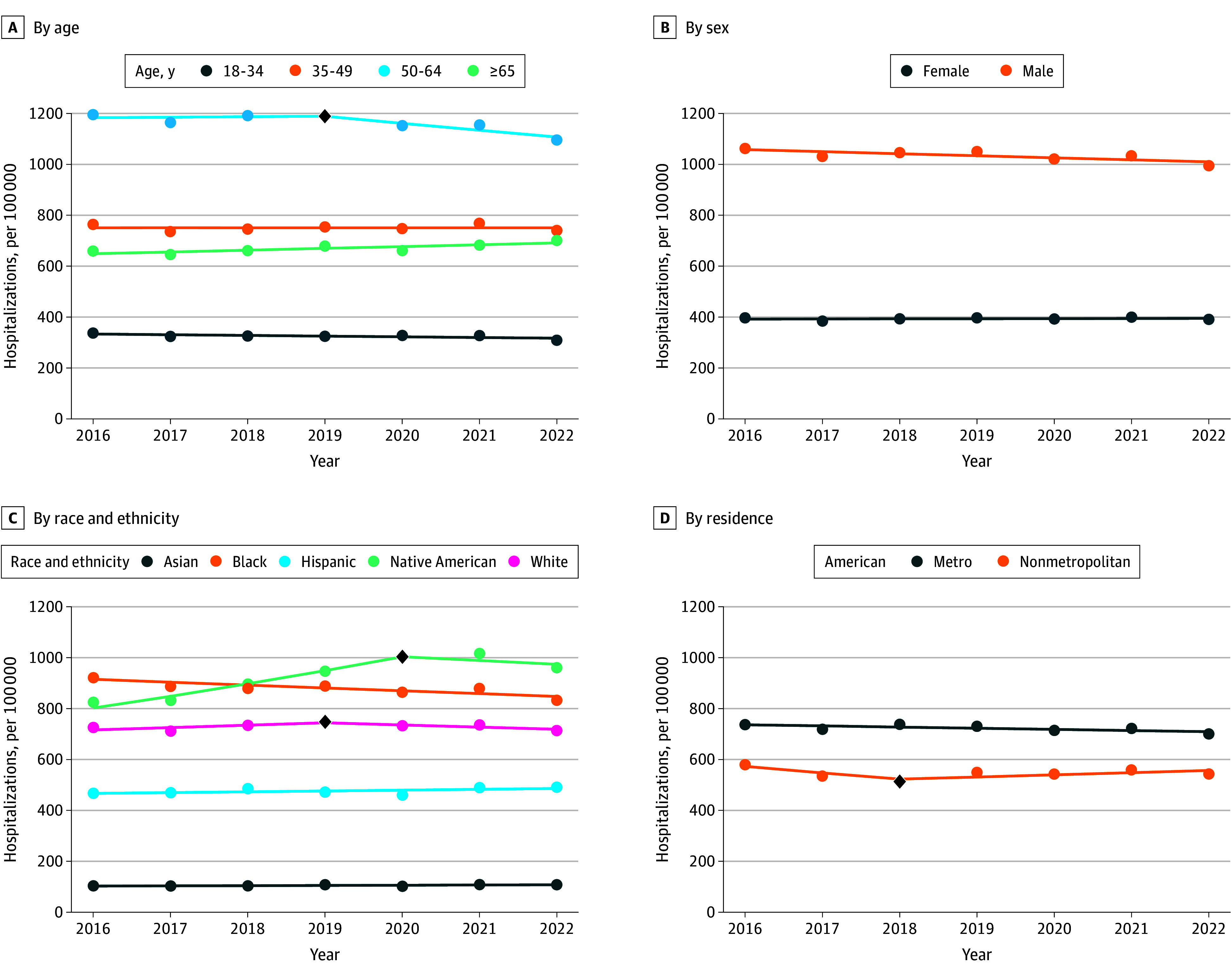
Trends in Rates of Alcohol-Related Hospitalizations by Demographic Subgroups Segments indicate annual percentage changes derived from joinpoint regressions. A single segement (0 joinpoints) indicates a consistent rate from 2016 to 2022, and a joinpoint indicated by a black diamond represents a change in direction or magnitude. Additional data can be found in eTable 13 in Supplement 1.

**Figure 3.**
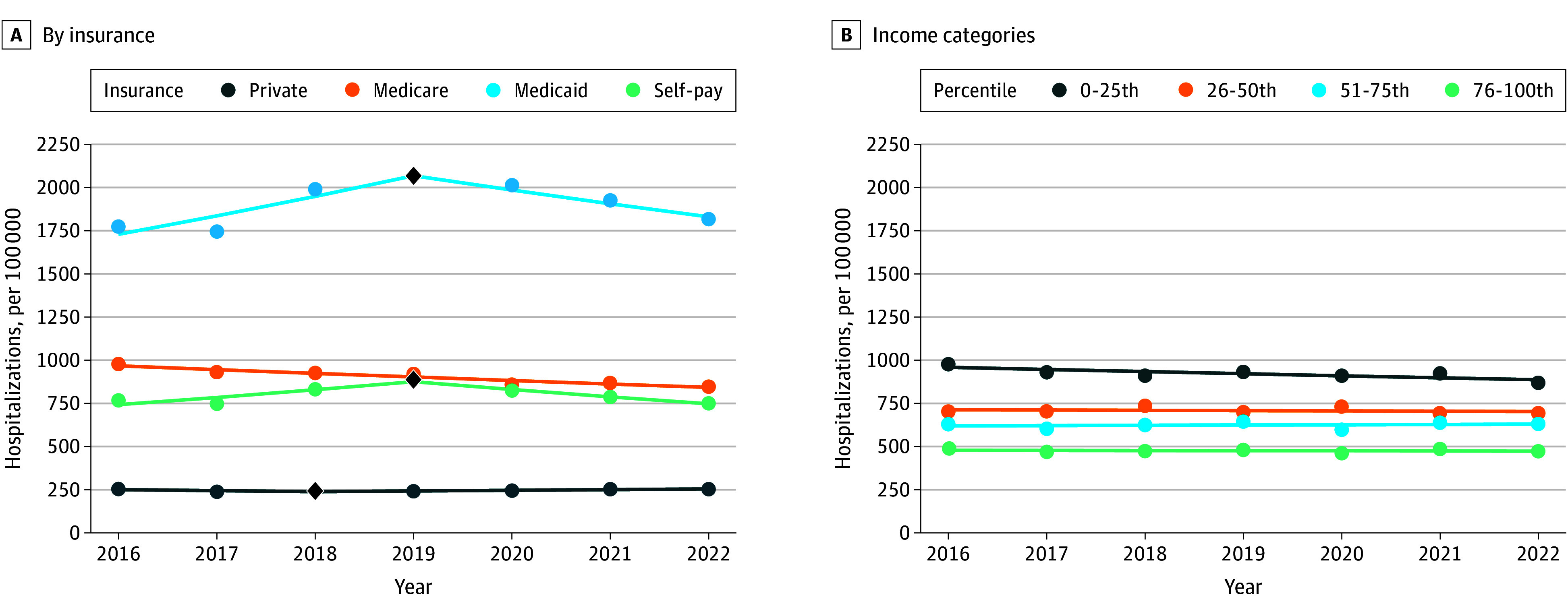
Trends in Rates of Alcohol-Related Hospitalizations by Socioeconomic Subgroups Segments indicate annual percentage changes derived from joinpoint regressions. A single segement (0 joinpoints) indicates a consistent rate from 2016 to 2022, and a joinpoint indicated by a black diamond represents a change in direction or magnitude. Additional data can be found in eTable 13 in Supplement 1.

When stratified by demographics across all alcohol-related hospitalizations, rates per 100 000 among ages 50 to 64 years decreased from 2292 in 2019 to 2096 in 2022 (APC, −2.35; 95% CI, −5.50 to −0.30), but there were no other significant changes across age or sex categories (Figure 2). Rates per 100 000 among Black adults decreased from 921 in 2016 to 832 in 2022 (APC, −1.27; 95% CI, −2.01 to −0.23). There were no significant differences in rates across metropolitan residence groups. Rates differed across census divisions but were largely stable with some exceptions, including decreased rates in the Middle Atlantic division from 2016 to 2022 (APC, −2.51; 95% CI, −3.82 to −1.20) (eFigure in Supplement 1). Regarding economic factors, rates per 100 000 among Medicaid beneficiaries increased from 1775 in 2016 to 2069 in 2019 (APC, 6.15; 95% CI, 3.50 to 10.33) before decreasing to 1818 in 2022 (APC, −4.00; 95% CI, −11.86 to −1.22) (Figure 3). Rates decreased in the Medicare group from 2016 to 2022 (APC, −2.28; 95% CI, −2.91 to −1.66) and increased in the self-pay group from 2016 to 2019 (APC, 5.64; 95% CI, 2.58 to 10.12) before decreasing from 2019 to 2022 (APC, −5.14; 95% CI, −8.89 to −2.40). There were no differences across income groups.

Trends by demographic and socioeconomic factors differed across types alcohol-related hospitalizations (eTables 4 to 9 in Supplement 1). Rates of primary AUD hospitalizations from 2016 to 2022 increased among adults age 65 years or older (APC, 3.84; 95% CI, 2.69 to 5.04), those who were Hispanic (APC, 3.26; 95% CI, 1.01 to 5.91) or Native American (APC, 6.63; 95% CI, 4.91 to 8.56), and in the West South Central (APC, 3.62; 95% CI, 1.74 to 5.81) and Pacific regions (APC, 2.83; 95% CI, 1.48 to 4.42). Rates of primary alcohol-related medical complication hospitalizations from 2016 to 2022 increased across all census divisions except New England. Rates also increased among ages 35 to 49 years (APC, 4.98; 95% CI, 3.30 to 6.73) and 50 to 64 years (APC, 2.21; 95% CI, 0.63 to 3.85), and Asian (APC, 5.01; 95% CI, 2.47 to 8.11), Hispanic (APC, 5.26; 95% CI, 3.04 to 7.79), and Native American adults (APC, 7.21; 95% CI, 3.91 to 10.64). Rates of primary alcohol-related medical complication hospitalizations also increased for those with private insurance (APC, 5.91; 95% CI, 3.81 to 8.10) and with higher income (51st to 75th percentile: APC, 4.47; 95% CI, 2.92 to 6.08; 65th to 100th percentile: APC, 5.00; 95% CI, 3.69 to 6.35). Rates of secondary AUD hospitalizations did not consistently increase for any group from 2016 to 2022.

### Outcomes of Alcohol-Related Hospitalizations

Among all alcohol-related hospitalizations, in-hospital mortality increased from 2.4% (95% CI, 2.3% to 2.5%) in 2016 to 3.1% (95% CI, 3.0% to 3.2%) in 2022 (*P* < .001) (Table 2). Length of stay also increased from 5.6 (95% CI 5.6-5.7) days in 2016 to 6.2 (95% CI 6.1-6.3) days in 2022 (*P* < .001). Average cost per hospitalization increased even after accounting for inflation, and overall, alcohol-related hospitalizations contributed to $32.6 billion in health care costs in 2022. Elixhauser scores increased from a mean of 22.3 (95% CI, 22.1-22.5) in 2016 to 23.7 (95% CI 23.5 to 23.9) in 2022 (*P* < .001), and self-directed discharges increased from 5.0% (95% CI, 4.8 to 5.2) in 2016 to 6.3% (95% CI 6.1% to 6.5%) in 2022 (*P* < .001). Hospitalization outcomes among diagnosis subgroups were consistent with the main analysis (eTables 10 to 12 in Supplement 1), although primary AUD hospitalizations had a low rate of in-hospital mortality 0.2% (95% CI, 0.1% to 0.2%) to 0.3% (95% CI, 0.2% to 0.3%) (*P* < .001).

**Table 2.** Trends in Hospitalization Outcomes During Alcohol-Related Hospitalizations, National Inpatient Sample 2016 to 2022

Outcome	Outcome during year (95% CI)							*P* value^a^
	2016	2017	2018	2019	2020	2021	2022	
In-hospital mortality, %	2.4 (2.3-2.5)	2.4 (2.4-2.5)	2.4 (2.3-2.5)	2.4 (2.3-2.5)	2.8 (2.8-2.9)	3.2 (3.1-3.3)	3.1 (3.0-3.2)	<.001
LOS, mean d	5.6 (5.6-5.7)	5.6 (5.5-5.6)	5.6 (5.5-5.6)	5.6 (5.6-5.7)	5.7 (5.7-5.8)	6.0 (5.9-6.0)	6.2 (6.1-6.3)	<.001
Mean cost per hospitalization, thousands, USD	12.5 (12.3-12.8)	12.8 (12.5-13.1)	13.1 (12.8-13.3)	14.0 (13.7-14.3)	15.4 (15.1-15.7)	16.4 (16.1-16.7)	17.9 (17.5-18.3)	<.001
Mean cost per hospitalization, thousands, 2016 USD	12.5 (12.3-12.8)	12.5 (12.3-12.8)	12.5 (12.3-12.8)	13.1 (12.8-13.4)	14.3 (14.0-14.5)	14.5 (14.2-14.8)	14.7 (14.4-15.0)	<.001
Elixhauser Index, mean	22.3 (22.1-22.5)	22.8 (22.6-23.0)	23.3 (23.1-23.5)	23.7 (23.5-23.9)	24.4 (24.2-24.6)	24.1 (23.9-24.3)	23.7 (23.5-23.9)	<.001
Discharge disposition, %^b^								
Self-directed discharge	5.0 (4.8-5.2)	5.3 (5.1-5.5)	5.5 (5.3-5.7)	5.6 (5.4-5.9)	6.3 (6.1-6.5)	6.5 (6.3-6.7)	6.3 (6.1-6.5)	<.001
Home	75.5 (75.2-75.9)	75.0 (74.7-75.3)	74.7 (74.4-75.0)	74.4 (74.1-74.7)	74.4 (74.1-74.7)	73.5 (73.2-73.8)	74.1 (73.8-74.4)	<.001
Facility	14.5 (14.3-14.8)	14.7 (14.5-15.0)	14.9 (14.6-15.1)	15.0 (14.8-15.3)	14.0 (13.8-14.2)	14.4 (14.1-14.6)	14.2 (13.9-14.4)	<.001
Other	2.5 (2.4-2.7)	2.5 (2.4-2.6)	2.6 (2.5-2.7)	2.6 (2.5-2.7)	2.5 (2.4-2.6)	2.4 (2.3-2.4)	2.3 (2.3-2.4)	<.001

Abbreviations: LOS, length of stay; USD, US dollars.

^a^ *P* value determined using univariable regressions fitted to the specific outcomes (logistic regression for mortality and discharge disposition, negative binomial for LOS, and linear for cost and Elixhauser).

^b^ Discharge disposition percentages calculated using total hospitalizations as a denominator, including those with in-hospital mortality.

## Discussion

In this nationally representative sample, we found overall stable trends in alcohol-related hospitalizations from 2016 to 2022 but rising rates of hospitalizations for primary alcohol-related medical complications. During this period, rates of inpatient mortality, length of stay, and patient complexity have increased, contributing to increasing inflation-adjusted costs and $32.6 billion in health care spending in 2022 alone. These findings highlight the need for in-hospital interventions to improve AUD outcomes and expansion of outpatient AUD treatment access to mitigate alcohol-related morbidity before progression to hospitalization. More broadly, implementation of evidence-based alcohol control policies, such as increased alcohol taxation and minimum unit pricing, are needed.^22^

Our finding of rising annual rates of primary AUD (up to 2019) and primary alcohol-related medical complication hospitalizations from 2016 to 2022 is consistent with prior trends.^9^ However, we did not find any changes in overall alcohol-related hospitalizations due to a concurrent reduction in secondary alcohol-related hospitalizations, which was largely driven by a decrease in hospitalizations with a primary psychiatric diagnosis. The reduction in secondary alcohol-related hospitalizations could reflect changes in coding practices or greater recognition of AUD as a primary cause of hospitalizations. Despite decreasing rates, secondary alcohol-related diagnoses represented the vast majority of alcohol-related hospitalizations. These findings suggest an opportunity to prioritize hospital-based treatment initiation in this group, given that patients with secondary alcohol-related diagnoses are less likely to receive treatment.^8^ Our findings of increased inpatient mortality, consistent with the known rise in general alcohol-related mortality in recent years,^11^ represent a change in trends from a prior study that found decreasing rates of inpatient mortality during AUD hospitalizations from 1998 to 2016.^9^ Research is needed to identify causes of in-hospital deaths in this population. A potential contributor is our finding of increased patient complexity, which has also been observed across general inpatient populations.^23^ Recently, increasing inpatient mortality has also been observed for ischemic stroke and gastrointestinal bleeds.^24,25^ Stable alcohol-related hospitalization rates in light of rising rates of alcohol-related mortality^11^ and emergency department visits^26^ suggest a possible change in the threshold to admit patients with AUD such that only patients with more severe illness are hospitalized.

Our results challenged the idea that the COVID-19 pandemic contributed to increases in alcohol-related hospitalizations. While prior studies using NIS found increased hospitalizations due to alcohol-related liver disease between 2019 and 2020,^27,28,29^ these could be related to long-standing trends that preceded the pandemic.^13^ A prior study that used commercial claims and accounted for baseline trends found increases in high acuity alcohol-related hospitalizations among women.^12^ However, private insurance represented less than a one-fourth of alcohol-related hospitalizations in our nationally representative sample. While alcohol use and alcohol-related harms have increased during the COVID-19 pandemic, it remains to be seen whether these changes will be offset by shifting admission thresholds, particularly for complications with longer latency periods.^10,11^

Our findings of increasing rates of primary AUD hospitalizations among older adults and increasing rates of primary AUD and primary alcohol-related medical complication hospitalizations among Hispanic adults are consistent with trends observed in prior studies using Medicare claims and NIS.^30,31^ We extend prior studies of trends in alcohol-related hospitalizations across demographics by describing markedly rising rates of primary AUD hospitalizations among Native American and primary AUD and primary alcohol related medical complication hospitalizations among Native American and Asian adults. These groups may face unique disparities in access to evidence-based AUD treatment.^8,32,33^ The increase in hospitalizations among patients who are Hispanic and Native American is particularly notable because Hispanic adults are more likely to experience inpatient hepatic complications compared to other patients with AUD,^34^ and Native Americans have the highest overall rate of alcohol-attributed mortality.^35^

Our findings suggest the need for clinical interventions, implementation trials, and policy-level changes to reduce alcohol-related hospitalizations and improve patient outcomes. Culturally-tailored interventions and community-based participatory research are needed to improve outcomes among Hispanic and Native American populations.^36^ Furthermore, our findings of increasing rates of self-directed discharges, which could be associated with undertreated withdrawal or stigma^37^ suggest opportunities to improve inpatient care delivery and AUD treatment engagement. Potential strategies include expansion of addiction consult services^38^ and hospitalist training through implementation facilitation.^39^

Because Medicaid had the highest rate of alcohol-related hospitalizations across payers, efforts to improve alcohol-related outcomes should be supported by comprehensive AUD treatment coverage to mitigate potential treatment barriers.^40^ The decreased rate of Medicaid hospitalizations we observed from 2019 to 2022 was likely driven by an increased number of Medicaid beneficiaries during this time period due to COVID-19 pandemic-related continuous enrollment policies rather than a true reduction in alcohol-related hospitalizations.^41^ More broadly, alcohol control policies, such as increased alcohol taxation and minimum unit pricing, which have been shown to reduce per capita alcohol consumption, should be expanded to reduce population-level alcohol-related morbidity and mortality.^22^

### Limitations

Our study was limited by potential misclassification of *ICD-10* codes, including identification of AUD and alcohol-related hospitalizations, which may be undercounted.^42^ Research is needed to validate the identification of AUD hospitalizations using administrative data. Further, given that alcohol use can contribute to hospitalizations through diseases that are not directly alcohol-related (eg, atrial fibrillation and pneumonia)^16^ and nonadherence to other medical treatment, it is difficult to precisely identify hospitalizations, which are alcohol-related using ICD codes alone. We tried to account for this by describing trends in a subgroup of secondary alcohol-related diagnosis hospitalizations. Last, while we evaluated trends across primary payers, we were unable to account for the presence of more than 1 insurance.

## Conclusions

In this serial cross-sectional study of nationally representative administrative claims from 2016-2022, the rate of alcohol-related hospitalizations remained stable, with increasing rates of inpatient mortality and length of stay, potentially driven by increased patient complexity. Expansion of evidence-based policies to reduce alcohol consumption and AUD treatment engagement is needed to improve patient outcomes and reduce the $32.6 billion in health care costs incurred by alcohol-related hospitalizations.

## Notes

Supplement 1. **eTable 1.** Identification of alcohol-related hospitalizations **eTable 2**. Primary discharge diagnoses among patients with primary alcohol-related complications **eTable 3.** Primary discharge diagnoses among patients with secondary alcohol-related diagnosis hospitalizations **eFigure. **Trends in alcohol-related hospitalizations stratified by census division **eTable 4. **Trends in primary AUD hospitalizations by sociodemographic categories **eTable 5. **Trends in primary AUD hospitalizations by sociodemographic categories **eTable 6. **Rates of primary alcohol-related medical complication hospitalizations by sociodemographic categories **eTable 7. **Trends in primary alcohol-related medical complication hospitalizations by sociodemographic categories **eTable 8. **Rates of secondary alcohol-related diagnosis hospitalizations by sociodemographic categories **eTable 9. **Trends in secondary alcohol-related diagnosis hospitalizations by sociodemographic categories **eTable 10. **Trends in hospitalization outcomes during primary AUD hospitalizations **eTable 11.** Trends in hospitalization outcomes during primary alcohol-related medical complication hospitalizations **eTable 12.** Trends in hospitalization outcomes during secondary alcohol-related diagnosis hospitalizations **eTable 13. **Trends in Rates of Overall Alcohol-Related Hospitalizations by Sociodemographic Subgroups

Supplement 2.
Data Sharing Statement
