# Supplementary material for: Alcohol-Related Hospitalizations From 2016 to 2022
Source: JAMA Netw Open. 2025 Dec 23;8(12):e2550589. doi: 10.1001/jamanetworkopen.2025.50589 (PMC12728657; doi:10.1001/jamanetworkopen.2025.50589)
Supplement: Supplement 1. — eTable 1. Identification of alcohol-related hospitalizations eTable 2. Primary discharge diagnoses among patients with primary alcohol-related complications eTable 3. Primary discharge diagnoses among patients with secondary alcohol-related diagnosis hospitalizations eFigure. Trends in alcohol-related hospitalizations stratified by census division eTable 4. Trends in primary AUD hospitalizations by sociodemographic categories eTable 5. Trends in primary AUD hospitalizations by sociodemographic categories eTable 6. Rates of primary alcohol-related medical complication hospitalizations by sociodemographic categories eTable 7. Trends in primary alcohol-related medical complication hospitalizations by sociodemographic categories eTable 8. Rates of secondary alcohol-related diagnosis hospitalizations by sociodemographic categories eTable 9. Trends in secondary alcohol-related diagnosis hospitalizations by sociodemographic categories eTable 10. Trends in hospitalization outcomes during primary AUD hospitalizations eTable 11. Trends in hospitalization outcomes during primary alcohol-related medical complication hospitalizations eTable 12. Trends in hospitalization outcomes during secondary alcohol-related diagnosis hospitalizations eTable 13. Trends in Rates of Overall Alcohol-Related Hospitalizations by Sociodemographic Subgroups [file jamanetwopen-e2550589-s001.pdf]

Supplemental Online Content

Bernstein EY, Wilson LM, Kruse GR, Edelman EJ, Herzig SJ, Anderson TS. Alcohol-related hospitalizations from 2016 to 2022. *JAMA Netw Open*. 2025;8(12):e2550589. doi:10.1001/jamanetworkopen.2025.50589

eTable 1. Identification of alcohol-related hospitalizations

eTable 2. Primary discharge diagnoses among patients with primary alcohol-related complications

eTable 3. Primary discharge diagnoses among patients with secondary alcohol-related diagnosis hospitalizations

eFigure. Trends in alcohol-related hospitalizations stratified by census division

eTable 4. Trends in primary AUD hospitalizations by sociodemographic categories

eTable 5. Trends in primary AUD hospitalizations by sociodemographic categories

eTable 6. Rates of primary alcohol-related medical complication hospitalizations by sociodemographic categories

eTable 7. Trends in primary alcohol-related medical complication hospitalizations by sociodemographic categories

eTable 8. Rates of secondary alcohol-related diagnosis hospitalizations by sociodemographic categories

eTable 9. Trends in secondary alcohol-related diagnosis hospitalizations by sociodemographic categories

eTable 10. Trends in hospitalization outcomes during primary AUD hospitalizations

eTable 11. Trends in hospitalization outcomes during primary alcohol-related medical complication hospitalizations

eTable 12. Trends in hospitalization outcomes during secondary alcohol-related diagnosis hospitalizations

eTable 13. Trends in Rates of Overall Alcohol-Related Hospitalizations by Sociodemographic Subgroups

This supplemental material has been provided by the authors to give readers additional information about their work.

© 2025 Bernstein EY et al. *JAMA Network Open*.

eTable 1. Identification of alcohol-related hospitalizations

Diagnosis	ICD-10 code
AUD	
Alcohol use/abuse/dependence	F10.1x, F10.2x, F10.9x excluding "in remission" specifiers (F10.11, F10.21, F10.91)
Alcohol-related medical complications	
Alcohol-related polyneuropathy	G62.1
Degeneration of nervous system due to alcohol	G31.2
Alcohol-related myopathy	G72.1
Alcohol-related cardiomyopathy	I42.6
Alcohol-related gastritis	K29.2
Alcohol-related liver disease	K70.0-K70.4, K70.9
Alcohol-induced acute pancreatitis	K85.2
Alcohol-induced chronic pancreatitis	K86.0

© 2025 Bernstein EY et al. *JAMA Network Open*.

eTable 2. Counts of primary discharge diagnoses among patients with primary alcohol-related complications

ICD-10 diagnoses	2016	2017	2018	2019	2020	2021	2022	Absolute change 2016-2022
Degeneration of nervous system due to alcohol	2,675	2,565	1,955	1,790	1,775	1,560	1,475	-1,200
Alcohol-associated polyneuropathy	860	900	1,145	1,045	1,355	1,585	1,395	535
Alcohol-associated myopathy	235	160	160	205	260	195	185	-50
Alcohol-associated cardiomyopathy	680	660	630	520	620	590	580	-100
Alcohol-associated gastritis	9,055	8,890	8,880	8,545	8,770	8,385	7,185	-1,870
Alcohol-associated gastritis without bleeding	6,520	5,960	5,950	5,490	5,775	5,540	4,555	-1,965
Alcohol-associated gastritis with bleeding	2,535	2,930	2,930	3,055	2,995	2,845	2,630	95
Alcohol-associated liver disease	91,450	98,490	104,915	110,955	121,130	138,855	136,040	44,590
Alcohol-associated fatty liver	190	195	190	175	240	145	170	-20
Alcohol-associated hepatitis without ascites	9,650	10,050	11,450	11,385	12,895	12,900	12,280	2,630
Alcohol-associated hepatitis with ascites	6,360	6,520	7,320	7,680	9,050	9,900	8,930	2,570
Alcohol-associated fibrosis and sclerosis of liver	25	5	0	20	20	10	5	-20
Alcohol-associated cirrhosis of liver without ascites	12,395	14,025	14,760	14,845	15,495	17,320	18,590	6,195
Alcohol-associated cirrhosis of liver with ascites	48,300	52,310	55,210	60,485	67,125	80,180	81,970	33,670
Alcohol-associated hepatic failure without coma	12,390	14,010	14,795	15,275	15,150	17,065	12,835	445
Alcohol-associated hepatic failure with coma	1,455	590	465	365	420	470	385	-1,070
Alcohol-associated liver disease, unspecified	685	785	725	725	735	865	875	190
Alcohol-associated pancreatitis	72,790	76,580	76,445	78,105	82,110	81,005	75,675	2,885
Alcohol induced acute pancreatitis	52,055	5	0	0	0	0	0	-52,055
Alcohol induced acute pancreatitis without necrosis or infection	17,125	70,655	70,030	71,600	74,785	73,220	68,350	51,225
Alcohol induced acute pancreatitis with uninfected necrosis	610	2,495	3,345	3,400	4,130	4,765	4,325	3,715
Alcohol induced acute pancreatitis with infected necrosis	175	510	545	605	790	710	695	520
Alcohol induced chronic pancreatitis	2,825	2,915	2,525	2,500	2,405	2,310	2,305	-520

© 2025 Bernstein EY et al. *JAMA Network Open*.

eTable 3. Counts of primary discharge diagnoses among patients with secondary alcohol-related diagnosis hospitalizations

Diagnosis group	2016	2017	2018	2019	2020	2021	2022	Absolute change 2016-2022
Psychiatric	283,545	267,285	261,875	250,110	225,865	211,425	199,590	-83,955
Circulatory	191,015	185,135	190,890	194,620	183,625	194,070	192,880	1,865
Injury	180,835	170,355	178,265	179,655	185,405	183,360	182,165	1,330
Digestive	193,230	185,215	185,300	188,100	183,770	188,000	181,405	-11,825
Infectious	102,495	106,010	117,445	120,795	149,690	163,385	156,720	54,225
Endocrine	62,160	64,500	69,225	73,005	78,030	80,585	81,640	19,480
Respiratory	95,120	90,765	91,510	91,890	80,580	68,945	70,545	-24,575
Nervous system	50,455	48,775	48,865	50,110	50,870	51,360	51,375	920
Genitourinary	45,685	44,120	46,865	47,645	46,480	46,660	47,080	1,395
Musculoskeletal	41,495	35,775	35,755	35,270	31,615	30,515	30,040	-11,455
Oncologic	30,810	29,140	29,110	29,385	26,280	27,975	27,860	-2,950
Other	27,155	23,410	23,055	21,660	21,595	21,715	21,200	-5,955
Skin	26,790	25,745	24,700	23,705	21,385	21,675	20,675	-6,115
Hematologic	14,580	14,155	14,175	14,770	15,105	16,060	15,150	570
Factors influencing health status	4,985	4,675	4,530	5,120	5,405	5,775	5,595	610
Pregnancy	4,365	3,575	4,045	4,090	3,690	3,620	3,895	-470
Congenital	770	755	765	720	730	720	1,020	250
Eye	835	705	790	870	730	800	730	-105
Ear	585	565	610	490	565	500	635	50
Perinatal			5	5	5			n/a

Diagnoses grouped using Healthcare Cost and Utilization Project Clinical Classification Software Refined

© 2025 Bernstein EY et al. *JAMA Network Open*.

eFigure. Trends in rates of alcohol-related hospitalizations stratified by census division

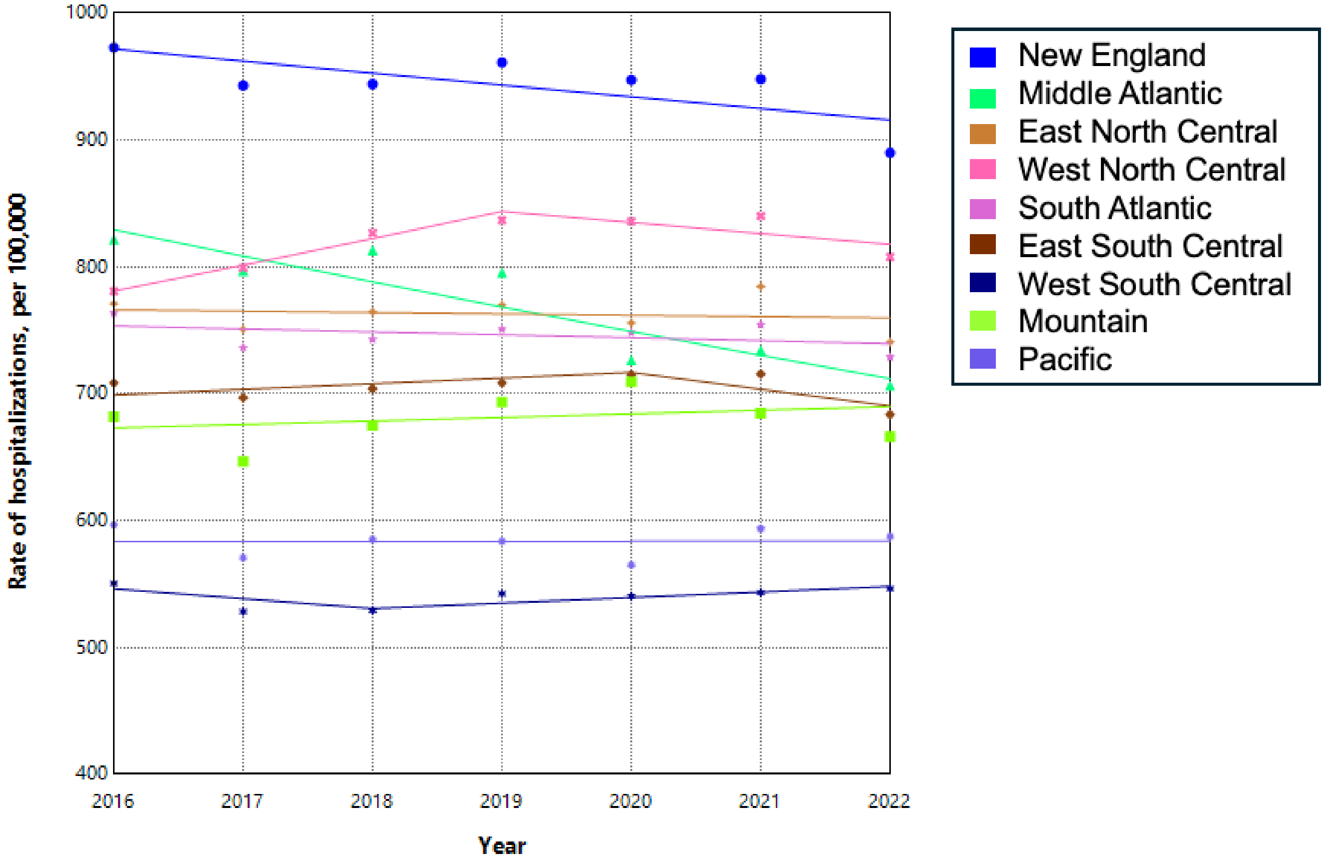

Annual percent changes (APCs) derived from joinpoint regressions where a single segment (0 joinpoints) indicates a consistent rate from 2016-2022 and two segments (1 joinpoint) indicate a change in direction or magnitude: New England, 2016-2022 APC -0.98 (95% CI -2.49, 0.70); Middle Atlantic, 2016-2022 APC -2.51 (95% CI -3.82, -1.20); East North Central, 2016-2022 APC -0.14 (95% CI -0.98, 0.70); West North Central, 2016-2019 APC 2.61 (95% CI 1.44, 5.17), 2019-2022 APC -1.04 (95% CI -2.94, 0.04); South Atlantic, 2016-2022 APC -0.31 (95% CI -1.25, 0.68); East South Central, 2016-2020 APC 0.63 (95% CI -0.14, 2.58), 2020-2022 APC -1.87 (95% CI -3.34, -0.22); West South Central, 2016-2018 APC -1.43 (95% CI -2.79, 0.16), 2018-2022 APC 0.81 (95% CI 0.07, 2.23); Mountain, 2016-2022 APC 0.41 (95% CI -1.67, 2.54); Pacific, 2016-2022 APC 0.01 (95% CI -1.24, 1.37)

© 2025 Bernstein EY et al. *JAMA Network Open*.

eTable 4. Rates of primary AUD hospitalizations by sociodemographic categories

Sociodemographic category	Annual rates per 100,000						
	**2016**	**2017**	**2018**	**2019**	**2020**	**2021**	**2022**
Age							
18-34	66.35	65.90	70.08	73.74	72.46	76.31	72.22
35-49	167.55	170.04	175.11	182.25	176.22	186.30	179.72
50-64	182.56	185.93	192.07	198.83	188.91	189.79	178.37
65+	48.71	52.03	54.32	56.95	56.61	61.17	61.30
Sex							
Female	59.63	60.21	63.55	65.65	62.98	65.66	62.89
Male	177.55	180.17	184.95	192.04	184.84	191.07	181.75
Race and ethnicity							
Asian	13.44	13.86	15.13	16.75	15.43	17.53	15.50
Black	113.59	113.16	113.52	116.63	107.99	114.69	107.24
Hispanic	68.97	73.08	78.83	82.43	76.47	85.07	85.21
Native	118.62	123.99	133.62	146.63	153.44	171.85	166.99
White	126.37	129.84	136.33	142.05	138.69	141.77	134.45
Residence							
Metropolitan	122.36	124.53	130.87	133.83	128.09	132.62	127.33
Non-metropolitan	77.38	76.95	72.36	77.72	77.27	79.86	75.93
Census division							
New England	217.66	226.26	225.85	223.45	223.24	230.94	211.81
Middle Atlantic	194.27	193.99	209.34	214.85	181.64	180.68	171.77
East North Central	128.41	130.87	137.42	141.32	136.25	145.83	134.39
West North Central	137.17	147.38	151.17	157.88	161.22	166.77	158.64
South Atlantic	105.26	106.96	104.38	110.70	110.32	115.23	108.24
East South Central	96.97	94.47	93.53	90.45	96.60	100.06	100.44
West South Central	63.04	59.63	61.97	68.13	68.61	71.53	74.09
Mountain	98.01	103.14	112.28	119.57	122.19	123.02	115.50
Pacific	78.24	79.23	83.34	87.88	85.98	91.72	91.47
Payer							
Private	45.66	44.60	45.61	46.34	43.84	46.58	46.36
Medicare	99.33	99.33	99.17	100.93	94.68	97.96	92.16
Medicaid	333.82	350.71	415.71	450.04	433.63	424.67	399.98
Self	154.55	151.16	170.87	184.93	169.76	163.81	153.98
Income percentile							
0-25th	136.92	134.98	133.25	119.35	134.49	142.64	132.71
26-50th	109.68	115.32	120.70	119.35	125.68	118.45	116.40
51-75th	105.38	108.47	114.21	122.26	111.02	122.83	118.34
76-100th	100.30	99.66	105.42	106.63	99.84	105.39	102.85

© 2025 Bernstein EY et al. *JAMA Network Open*.

eTable 5. Trends in rates of primary AUD hospitalizations by sociodemographic categories

Sociodemographic category	Segment	Lower endpoint	Upper endpoint	APC (95% CI)	P-value
**Age**					
18-34	1	2016	2022	2.05 (-0.90, 5.45)	0.17
35-49	1	2016	2022	1.41 (-0.06, 3.10)	0.06
50-64	1	2016	2019	2.82 (1.15, 5.27)	<0.01
	2	2019	2022	-2.99 (-5.15, -1.40)	<0.01
65+	1	2016	2022	3.84 (2.69, 5.04)	<0.01
**Sex**					
Female	1	2016	2019	3.20 (-0.87, 9.91)	0.14
	2	2019	2022	-0.80 (-6.39, 3.17)	0.52
Male	1	2016	2019	2.53 (-0.16, 7.16)	0.07
	2	2019	2022	-1.10 (-4.88, 1.62)	0.23
**Race and ethnicity**					
Asian	1	2016	2022	3.03 (-0.77, 7.98)	0.11
Black	1	2016	2022	-0.59 (-2.58, 1.73)	0.54
Hispanic	1	2016	2022	3.26 (1.01, 5.91)	0.01
Native	1	2016	2022	6.63 (4.91, 8.56)	<0.01
White	1	2016	2019	4.18 (2.40, 7.64)	<0.01
	2	2019	2022	-1.38 (-4.00, 0.27)	0.10
**Residence**					
Metropolitan	1	2016	2019	2.86 (1.16, 6.65)	0.01
	2	2019	2022	-1.15 (-4.41, 0.52)	0.19
Non-metropolitan	1	2016	2022	0.38 (-0.90, 1.79)	0.55
**Census division**					
New England	1	2016	2022	-0.13 (-1.27, 1.05)	0.85
Middle Atlantic	1	2016	2019	2.35 (-2.51, 11.66)	0.30
	2	2019	2022	-6.72 (-15.36, -2.05)	0.02
East North Central	1	2016	2022	1.10 (-1.67, 4.28)	0.34
West North Central	1	2016	2020	4.21 (1.15, 10.20)	0.01
	2	2020	2022	-1.00 (-6.28, 3.86)	0.72
South Atlantic	1	2016	2022	0.97 (-0.26, 2.34)	0.12
East South Central	1	2016	2019	-1.70 (-7.00, 2.62)	0.25
	2	2019	2022	3.64 (0.22, 8.30)	0.04
West South Central	1	2016	2022	3.62 (1.74, 5.81)	<0.01
Mountain	1	2016	2020	6.21 (4.81, 10.93)	<0.01
	2	2020	2022	-3.35 (-7.85, 1.21)	0.18
Pacific	1	2016	2022	2.83 (1.48, 4.42)	<0.01
**Payer**					
Private	1	2016	2022	0.40 (-0.58, 1.53)	0.40
Medicare	1	2016	2022	-1.04 (-2.62, 0.64)	0.20
Medicaid	1	2016	2019	11.26 (6.67, 18.38)	<0.01
	2	2019	2022	-3.68 (-10.06, 0.30)	0.07
Self	1	2016	2019	6.84 (3.31, 12.74)	<0.01
	2	2019	2022	-5.38 (-9.26, -2.43)	<0.01
**Income percentile**					
0-25th	1	2016	2022	0.20 (-1.61, 2.17)	0.79
26-50th	1	2016	2020	2.97 (1.41, 8.75)	0.02
	2	2020	2022	-3.81 (-8.52, -0.01)	0.048
51-75th	1	2016	2022	2.03 (-0.79, 5.27)	0.16
76-100th	1	2016	2022	0.49 (-1.90, 3.01)	0.57

APC, annual percent change; CI, confidence interval.
Segments are determined by joinpoint regressions. A single segment indicates a consistent rate from 2016-2022 while two segments indicate a change in direction or magnitude. Lower and upper endpoints describe the time periods for each segment.

© 2025 Bernstein EY et al. *JAMA Network Open*.

eTable 6. Rates of primary alcohol-related medical complication hospitalizations by sociodemographic categories

Sociodemographic category	Annual rates per 100,000						
	**2016**	**2017**	**2018**	**2019**	**2020**	**2021**	**2022**
Age							
18-34	30.11	31.42	32.75	36.10	40.18	41.71	40.56
35-49	94.25	98.44	101.50	104.69	115.24	124.77	121.28
50-64	119.44	125.71	127.75	128.30	131.59	143.20	132.42
65+	37.64	41.06	42.28	44.56	44.88	46.29	45.91
Sex							
Female	42.69	44.72	46.02	47.88	52.20	56.29	54.01
Male	98.44	103.44	105.48	107.89	112.44	119.76	113.59
Race and ethnicity							
Asian	12.01	14.15	14.34	14.48	14.94	16.36	16.90
Black	83.86	89.31	88.08	87.43	93.25	99.74	90.90
Hispanic	58.00	64.29	66.65	66.51	70.48	78.27	80.47
Native	93.55	101.30	104.37	109.13	127.39	152.71	130.31
White	68.06	71.33	73.78	77.79	82.09	86.63	81.76
Residence							
Metropolitan	71.81	76.18	78.61	79.78	84.44	90.16	86.30
Non-metropolitan	56.93	55.80	54.71	59.43	64.31	69.01	64.03
Census division							
New England	92.13	96.03	98.61	102.89	110.28	115.35	101.73
Middle Atlantic	66.36	69.69	71.71	73.34	73.21	82.37	76.85
East North Central	73.79	77.13	79.06	82.65	88.06	95.32	88.85
West North Central	69.42	72.78	78.06	80.25	84.19	90.55	86.57
South Atlantic	74.22	78.50	78.01	80.23	85.08	88.84	86.00
East South Central	70.01	72.94	74.84	78.98	88.04	90.82	83.64
West South Central	63.76	69.41	69.49	71.06	76.06	79.81	80.60
Mountain	76.00	75.96	79.47	84.29	90.66	98.30	93.31
Pacific	59.35	62.12	64.44	63.43	68.20	73.44	70.79
Payer							
Private	27.47	28.32	29.79	30.52	34.35	37.05	37.51
Medicare	68.34	71.25	70.10	70.58	67.11	71.10	64.12
Medicaid	189.64	199.95	225.28	241.10	256.27	263.40	244.99
Self	89.21	93.77	107.63	116.27	114.93	112.44	111.70
Income percentile							
0-25th	92.32	95.91	92.35	76.66	101.46	110.17	100.05
26-50th	70.33	75.26	79.14	76.66	86.83	86.77	84.94
51-75th	63.34	67.09	69.66	73.93	74.57	82.27	81.09
76-100th	47.19	48.31	51.36	53.50	56.74	62.45	60.52

© 2025 Bernstein EY et al. *JAMA Network Open*.

eTable 7. Trends in rates of primary alcohol-related medical complication hospitalizations by sociodemographic categories

Sociodemographic category	Segment	Lower endpoint	Upper endpoint	APC (95% CI)	P-value
**Age**					
18-34	1	2016	2020	7.88 (3.12, 16.21)	0.00
	2	2020	2022	1.97 (-4.61, 8.39)	0.30
35-49	1	2016	2022	4.98 (3.30, 6.73)	<0.01
50-64	1	2016	2022	2.21 (	<0.01
65+	1	2016	2019	5.44 (4.16, 7.64)	<0.01
	2	2019	2022	1.08 (-0.85, 2.29)	0.23
**Sex**					
Female	1	2016	2022	4.73 (3.14, 6.35)	<0.01
Male	1	2016	2022	2.88 (1.81, 3.96)	<0.01
**Race and ethnicity**					
Asian	1	2016	2022	5.01 (2.47, 8.11)	<0.01
Black	1	2016	2022	1.85 (-0.45, 4.36)	0.11
Hispanic	1	2016	2022	5.26 (3.04, 7.79)	<0.01
Native	1	2016	2022	7.21 (3.91, 10.64)	<0.01
White	1	2016	2020	5.16 (2.35, 9.74)	<0.01
	2	2020	2022	0.54 (-3.61, 5.19)	0.52
**Residence**					
Metropolitan	1	2016	2022	3.52 (1.83, 5.27)	<0.01
Non-metropolitan	1	2016	2022	3.38 (-0.78, 7.87)	0.11
**Census division**					
New England	1	2016	2020	5.08 (1.06, 13.57)	0.03
	2	2020	2022	-2.97 (-8.90, 4.16)	0.40
Middle Atlantic	1	2016	2022	2.90 (1.41, 4.42)	<0.01
East North Central	1	2016	2022	3.97 (2.64, 5.32)	<0.01
West North Central	1	2016	2022	4.33 (2.95, 5.75)	<0.01
South Atlantic	1	2016	2022	2.83 (0.94, 4.68)	<0.01
East South Central	1	2016	2022	4.40 (2.42, 6.59)	<0.01
West South Central	1	2016	2022	4.01 (2.80, 5.32)	<0.01
Mountain	1	2016	2022	4.65 (1.80, 7.22)	<0.01
Pacific	1	2016	2022	3.34 (2.24, 4.47)	<0.01
**Payer**					
Private	1	2016	2022	5.91 (3.81, 8.10)	<0.01
Medicare	1	2016	2022	-0.82 (-3.15, 1.54)	0.41
Medicaid	1	2016	2020	8.59 (6.78, 14.55)	<0.01
	2	2020	2022	-2.45 (-8.25, 2.95)	0.38
Self	1	2016	2019	9.73 (7.74, 11.65)	<0.01
	2	2019	2022	-1.46 (-3.31, 0.30)	0.08
**Income percentile**					
0-25th	1	2016	2022	2.18 (-1.71, 6.33)	0.30
26-50th	1	2016	2022	3.45 (0.35, 6.72)	0.03
51-75th	1	2016	2022	4.47 (2.92, 6.08)	<0.01
76-100th	1	2016	2022	5.00 (3.69, 6.35)	<0.01

APC, annual percent change; CI, confidence interval.
Segments are determined by joinpoint regressions. A single segment indicates a consistent rate from 2016-2022 while two segments indicate a change in direction or magnitude. Lower and upper endpoints describe the time periods for each segment.

© 2025 Bernstein EY et al. *JAMA Network Open*.

eTable 8. Rates of secondary alcohol-related diagnosis hospitalizations by sociodemographic categories

**Sociodemographic category**	Annual rates per 100,000 **2016**	**2017**	**2018**	**2019**	**2020**	**2021**	**2022**
Age							
18-34	241.17	226.60	222.89	214.79	215.05	209.37	196.40
35-49	501.87	467.00	468.73	467.08	455.68	457.24	438.78
50-64	893.11	852.75	871.87	863.88	831.90	821.70	785.06
65+	572.92	552.72	564.50	577.19	559.82	575.12	594.07
Sex							
Female	294.61	279.27	283.42	283.54	277.80	277.87	274.15
Male	786.40	746.78	755.03	749.97	723.15	722.53	699.10
Race and ethnicity							
Asian	78.22	75.19	74.12	76.83	71.64	74.94	75.86
Black	723.49	684.31	694.75	684.44	662.59	664.45	634.55
Hispanic	340.57	332.35	340.43	322.70	313.05	326.51	325.30
Native	612.57	606.88	641.17	691.05	725.01	692.07	663.32
White	531.71	510.40	523.94	528.34	511.70	507.46	497.66
Residence							
Metropolitan	542.58	518.14	528.42	516.97	501.58	499.13	486.64
Non-metropolitan	445.26	401.95	384.82	411.69	401.09	410.41	403.03
Census division							
New England	662.78	620.36	619.31	634.51	613.48	601.33	576.02
Middle Atlantic	560.90	533.04	531.79	507.21	471.58	471.09	457.65
East North Central	568.61	542.61	547.82	545.74	531.34	543.19	517.34
West North Central	573.95	578.82	597.21	598.31	590.20	582.50	562.43
South Atlantic	583.98	550.67	560.24	559.80	552.66	550.21	534.46
East South Central	541.30	529.42	535.53	538.84	530.24	524.56	499.40
West South Central	423.75	399.20	397.67	403.07	395.60	391.86	391.67
Mountain	507.78	467.42	483.05	489.41	496.53	463.15	457.11
Pacific	458.99	429.30	437.31	432.36	410.77	428.38	425.23
Payer							
Private	180.85	165.79	167.94	164.05	166.90	170.21	169.50
Medicare	810.25	760.22	756.79	749.43	697.54	698.99	690.64
Medicaid	1251.15	1195.14	1349.98	1377.54	1324.95	1238.52	1172.53
Self	522.57	502.66	554.03	590.56	539.95	510.77	484.78
Income percentile							
0-25th	747.80	699.49	684.68	503.16	674.22	671.45	636.44
26-50th	522.21	512.47	535.88	503.16	518.80	487.87	491.72
51-75th	461.37	427.99	440.96	449.05	411.50	433.69	432.13
76-100th	340.81	321.06	316.78	320.45	304.75	318.48	309.35

© 2025 Bernstein EY et al. *JAMA Network Open*.

eTable 9. Trends in rates of secondary alcohol-related diagnosis hospitalizations by sociodemographic categories

Sociodemographic category	Segment	Lower endpoint	Upper endpoint	APC (95% CI)	P-value
**Age**					
18-34	1	2016	2022	-2.85 (-4.06, -1.55)	<0.01
35-49	1	2016	2022	-1.66 (-2.47, -0.82)	<0.01
50-64	1	2016	2022	-1.81 (-2.64, -0.93)	<0.01
65+	1	2016	2022	0.64 (-0.95, 2.27)	0.37
**Sex**					
Female	1	2016	2022	-0.87 (-1.43, -0.28)	<0.01
Male	1	2016	2022	-1.64 (-2.43, -0.78)	<0.01
**Race and ethnicity**					
Asian	1	2016	2022	-0.40 (-1.84, 1.30)	0.69
Black	1	2016	2022	-1.78 (-2.54, -0.93)	<0.01
Hispanic	1	2016	2020	-1.85 (-4.36, -0.55)	0.02
	2	2020	2022	1.47 (-0.94, 3.76)	0.27
Native	1	2016	2020	4.85 (3.63, 6.91)	<0.01
	2	2020	2022	-3.86 (-6.20, -0.53)	0.02
White	1	2016	2022	-0.83 (-1.53, -0.12)	0.02
**Residence**					
Metropolitan	1	2016	2022	-1.60 (-2.21, -0.95)	<0.01
Non-metropolitan	1	2016	2018	-5.57 (-9.92, 0.23)	0.06
	2	2018	2022	1.22 (-1.99, 7.15)	0.25
**Census division**					
New England	1	2016	2022	-1.86 (-3.02, -0.53)	<0.01
Middle Atlantic	1	2016	2022	-3.42 (-4.11, -2.70)	<0.01
East North Central	1	2016	2022	-1.11 (-2.24, 0.05)	0.06
West North Central	1	2016	2019	1.66 (0.48, 4.99)	<0.01
	2	2019	2022	-2.05 (-4.85, -0.91)	<0.01
South Atlantic	1	2016	2022	-1.00 (-1.61, -0.37)	<0.01
East South Central	1	2016	2020	-0.11 (-1.16, 3.04)	0.92
	2	2020	2022	-3.07 (-5.35, -0.99)	<0.01
West South Central	1	2016	2022	-1.00 (-1.91, 0.00)	0.05
Mountain	1	2016	2022	-1.05 (-3.53, 1.42)	0.31
Pacific	1	2016	2022	-1.02 (-2.16, 0.21)	0.11
**Payer**					
Private	1	2016	2018	-3.89 (-6.91, -0.01)	0.049
	2	2018	2022	0.87 (-1.01, 4.52)	0.22
Medicare	1	2016	2022	-2.57 (-4.08, -1.04)	<0.01
Medicaid	1	2016	2019	4.46 (1.32, 10.76)	<0.01
	2	2019	2022	-5.23 (-9.83, -2.41)	<0.01
Self	1	2016	2019	4.55 (1.61, 9.02)	<0.01
	2	2019	2022	-5.86 (-9.64, -3.18)	<0.01
**Income percentile**					
0-25th	1	2016	2022	-2.07 (-6.57, 2.64)	0.39
26-50th	1	2016	2022	-1.07 (-2.15, -0.01)	0.048
51-75th	1	2016	2022	-0.84 (-2.45, 0.75)	0.29
76-100th	1	2016	2022	-1.21 (-3.07, 0.82)	0.22

APC, annual percent change; CI, confidence interval.
Segments are determined by joinpoint regressions. A single segment indicates a consistent rate from 2016-2022 while two segments indicate a change in direction or magnitude. Lower and upper endpoints describe the time periods for each segment.

© 2025 Bernstein EY et al. *JAMA Network Open*.

eTable 10. Trends in hospitalization outcomes during primary AUD hospitalizations

Outcome	2016	2017	2018	2019	2020	2021	2022	P-value [A]
In-hospital mortality, %	0.15	0.16	0.14	0.16	0.19	0.25	0.28	<0.01
LOS, mean days	4.58	4.59	4.57	4.50	4.49	4.62	4.68	<0.01
Average cost, USD	6,785	7,073	7,226	7,695	8,525	9,067	9,784	<0.01
Average cost, 2016 USD	6,785	6,926	6,907	7,224	7,906	8,031	8,023	<0.01
Total cost, billion USD	2.00	2.15	2.29	2.54	2.74	3.03	3.14	<0.01
Elixhauser index, mean	13.11	13.56	13.93	14.49	15.58	15.82	14.97	<0.01
Discharge disposition, [B] %								
Self-directed discharge	9.63	9.86	10.20	10.64	11.30	11.74	11.31	<0.01
Home	77.69	77.07	76.46	76.12	75.78	75.02	76.19	<0.01
Facility	10.77	11.01	11.37	11.34	10.98	11.33	10.57	0.79
Other	1.76	1.90	1.83	1.74	1.75	1.66	1.66	0.14

USD, United States Dollars; LOS, length of stay; CI, confidence interval
[A] P-value determined using univariable regressions fitted to the specific outcomes (logistic regression for mortality and discharge disposition, negative binomial for LOS, and linear for cost and Elixhauser)
[B] Discharge disposition percentages calculated using total hospitalizations as a denominator, including those with in-hospital mortality

© 2025 Bernstein EY et al. *JAMA Network Open*.

eTable 11. Trends in hospitalization outcomes during primary alcohol-related medical complication hospitalizations

Outcome	2016	2017	2018	2019	2020	2021	2022	P-value [A]
In-hospital mortality, %	3.06	3.00	2.84	2.81	3.28	3.68	3.87	<0.01
LOS, mean days	5.32	5.14	5.23	5.29	5.37	5.71	5.91	<0.01
Average cost, USD	12,290	12,483	12,868	13,866	15,285	16,684	18,318	<0.01
Average cost, 2016 USD	12,290	12,223	12,299	13,017	14,174	14,777	15,023	<0.01
Total cost, billion USD	2.16	2.34	2.49	2.77	3.27	3.83	4.03	<0.01
Elixhauser index, mean	24.32	24.41	24.71	25.24	25.49	25.36	25.03	<0.01
Discharge disposition, [B] %								
Self-directed discharge	4.75	5.35	5.30	5.33	5.96	6.09	5.63	<0.01
Home	80.83	80.49	80.67	80.26	80.61	79.33	79.96	<0.01
Facility	8.95	8.66	8.74	8.87	7.74	8.35	8.10	<0.01
Other	2.42	2.49	2.45	2.73	2.42	2.55	2.44	0.82

USD, United States Dollars; LOS, length of stay; CI, confidence interval

[A] P-value determined using univariable regressions fitted to the specific outcomes (logistic regression for mortality and discharge disposition, negative binomial for LOS, and linear for cost and Elixhauser)

[B] Discharge disposition percentages calculated using total hospitalizations as a denominator, including those with in-hospital mortality

© 2025 Bernstein EY et al. *JAMA Network Open*.

eTable 12. Trends in hospitalization outcomes during secondary alcohol-related diagnosis hospitalizations

Outcome	2016	2017	2018	2019	2020	2021	2022	P-value [A]
In-hospital mortality, %	2.82	2.90	2.86	2.88	3.42	3.87	3.66	<0.01
LOS, mean days	5.88	5.88	5.89	5.96	6.11	6.39	6.62	<0.01
Average cost, USD	13,812	14,191	14,520	15,530	17,072	18,234	19,875	<0.01
Average cost, 2016 USD	13,812	13,895	13,878	14,579	15,832	16,151	16,299	<0.01
Total cost, billion USD	18.49	18.37	19.19	20.56	22.16	23.77	25.39	<0.01
Elixhauser index, mean	24.03	24.76	25.31	25.78	26.46	26.07	25.67	<0.01
Discharge disposition, [B] %								
Self-directed discharge	3.97	4.20	4.41	4.45	5.05	5.29	5.14	<0.01
Home	74.37	73.75	73.38	73.06	73.09	72.09	72.56	<0.01
Facility	16.10	16.46	16.60	16.84	15.79	16.23	16.13	0.14
Other	2.73	2.69	2.75	2.76	2.65	2.51	2.51	<0.01

USD, United States Dollars; LOS, length of stay; CI, confidence interval
[A] P-value determined using univariable regressions fitted to the specific outcomes (logistic regression for mortality and discharge disposition, negative binomial for LOS, and linear for cost and Elixhauser)
[B] Discharge disposition percentages calculated using total hospitalizations as a denominator, including those with in-hospital mortality

© 2025 Bernstein EY et al. *JAMA Network Open*.

eTable 13. Trends in Rates of Overall Alcohol-Related Hospitalizations by Sociodemographic Subgroups

Sociodemographic category	Segment	Lower endpoint	Upper endpoint	APC (95% CI)	P-value
**Age**					
18-34	1	2016	2022	-0.85 (-2.45, 0.94)	0.31
35-49	1	2016	2022	-0.01 (-0.99, 1.09)	0.95
50-64	1	2016	2019	0.17 (-1.92, 3.58)	0.79
50-64	2	2019	2022	-2.35 (-5.50, -0.30)	0.02
65+	1	2016	2022	1.06 (-0.43, 2.62	0.17
**Sex**					
Female	1	2016	2022	0.12 (-0.77, 1.09)	0.73
Male	1	2016	2022	0.78 (-1.76, 0.30)	0.15
**Race and ethnicity**					
Asian	1	2016	2022	0.80 (-0.19, 1.98)	0.11
Black	1	2016	2022	-1.27 (-2.21, -0.23)	0.02
Hispanic	1	2016	2022	0.67 (-0.32, 1.78)	0.18
Native	1	2016	2020	5.76 (1.90, 13.72)	0.01
	2	2020	2022	-1.49 (-7.70, 4.73)	0.68
White	1	2016	2019	1.29 (0.14, 3.72)	0.04
	2	2019	2022	-1.16 (-3.43, -0.05)	0.04
**Residence**					
Metropolitan	1	2016	2022	-0.63 (-1.64, 0.49)	0.25
Non-metropolitan	1	2016	2018	4.48 (-11.02, 4.83)	0.23
	2	2018	2022	1.60 (-6.37, 9.86)	0.26
**Payer**					
Private	1	2016	2018	-2.31 (-7.50, 4.91)	0.36
	2	2018	2022	1.59 (-4.24, 7.44)	0.21
Medicare	1	2016	2022	-2.28 (-2.91, -1.66)	<0.01
Medicaid	1	2016	2019	6.15 (3.50, 10.33)	<0.01
	2	2019	2022	-4.00 (-11.86, -1.22)	<0.01
Self	1	2016	2019	5.64 (2.58, 10.12)	<0.01
	2	2019	2022	-5.14 (-8.89, -2.40)	<0.01
**Income percentile**					
0-25th	1	2016	2022	-1.29 (-5.35, 2.98)	0.54
26-50th	1	2016	2022	-0.23 (-1.19, 0.71)	0.61
51-75th	1	2016	2022	0.28 (-1.03, 1.58)	0.70
76-100th	1	2016	2022	-0.18 (-1.65, 1.41)	0.79

APC, annual percent change; CI, confidence interval.
Segments are determined by joinpoint regressions. A single segment indicates a consistent rate from 2016-2022 while two segments indicate a change in direction or magnitude. Lower and upper endpoints describe the time periods for each segment.

© 2025 Bernstein EY et al. *JAMA Network Open*.
