# Supplementary material for: Alcohol-Related Hospitalizations From 2016 to 2022
Source: JAMA Netw Open. 2025 Dec 23;8(12):e2550589. doi: 10.1001/jamanetworkopen.2025.50589 (PMC12728657; doi:10.1001/jamanetworkopen.2025.50589)
Supplement: Supplement 2. — Data Sharing Statement [file jamanetwopen-e2550589-s002.pdf]

Data Sharing Statement

Data
Data available: No

Additional Information
Explanation for why data not available: Data are managed by the Agency for Healthcare Research and Quality Healthcare Cost and Utilization Project.
